# Comparison of an automatic analysis and a manual analysis of conjunctival microcirculation in a sheep model of haemorrhagic shock

**DOI:** 10.1186/s40635-016-0110-5

**Published:** 2016-11-18

**Authors:** Philip-Helge Arnemann, Michael Hessler, Tim Kampmeier, Andrea Morelli, Hugo Karel Van Aken, Martin Westphal, Sebastian Rehberg, Christian Ertmer

**Affiliations:** 1 Department of Anaesthesiology, Intensive Care and Pain Therapy, Muenster University Hospital, Albert-Schweitzer-Campus 1, Building A1, 49149 Muenster, Germany; 2 Department of Cardiovascular, Respiratory, Nephrological, Anesthesiological and Geriatric Sciences, University of Rome, “La Sapienza”, Viale del Policlinico 155, 00161 Rome, Italy; 3 Department of Anaesthesiology, Greifswald University Hospital, Ferdinand-Sauerbruch-Straße, 17475 Greifswald, Germany

**Keywords:** Microcirculation, Analysis, Automatic, Manual, Haemorrhagic shock

## Abstract

**Background:**

Life-threatening diseases of critically ill patients are known to derange microcirculation. Automatic analysis of microcirculation would provide a bedside diagnostic tool for microcirculatory disorders and allow immediate therapeutic decisions based upon microcirculation analysis.

**Methods:**

After induction of general anaesthesia and instrumentation for haemodynamic monitoring, haemorrhagic shock was induced in ten female sheep by stepwise blood withdrawal of 3 × 10 mL per kilogram body weight. Before and after the induction of haemorrhagic shock, haemodynamic variables, samples for blood gas analysis, and videos of conjunctival microcirculation were obtained by incident dark field illumination microscopy. Microcirculatory videos were analysed (1) manually with AVA software version 3.2 by an experienced user and (2) automatically by AVA software version 4.2 for total vessel density (TVD), perfused vessel density (PVD) and proportion of perfused vessels (PPV). Correlation between the two analysis methods was examined by intraclass correlation coefficient and Bland-Altman analysis.

**Results:**

The induction of haemorrhagic shock decreased the mean arterial pressure (from 87 ± 11 to 40 ± 7 mmHg; *p* < 0.001); stroke volume index (from 38 ± 14 to 20 ± 5 ml·m^−2^; *p* = 0.001) and cardiac index (from 2.9 ± 0.9 to 1.8 ± 0.5 L·min^−1^·m^−2^; *p* < 0.001) and increased the heart rate (from 72 ± 9 to 87 ± 11 bpm; *p* < 0.001) and lactate concentration (from 0.9 ± 0.3 to 2.0 ± 0.6 mmol·L^−1^; *p* = 0.001). Manual analysis showed no change in TVD (17.8 ± 4.2 to 17.8 ± 3.8 mm*mm^−2^; *p* = 0.993), whereas PVD (from 15.6 ± 4.6 to 11.5 ± 6.5 mm*mm^−2^; *p* = 0.041) and PPV (from 85.9 ± 11.8 to 62.7 ± 29.6%; *p* = 0.017) decreased significantly. Automatic analysis was not able to identify these changes. Correlation analysis showed a poor correlation between the analysis methods and a wide spread of values in Bland-Altman analysis.

**Conclusions:**

As characteristic changes in microcirculation during ovine haemorrhagic shock were not detected by automatic analysis and correlation between automatic and manual analyses (current gold standard) was poor, the use of the investigated software for automatic analysis of microcirculation cannot be recommended in its current version at least in the investigated model. Further improvements in automatic vessel detection are needed before its routine use.

**Electronic supplementary material:**

The online version of this article (doi:10.1186/s40635-016-0110-5) contains supplementary material, which is available to authorized users.

## Background

Life-threatening diseases of critically ill patients are often accompanied by changes in microvascular perfusion [1–5], and the persistence of microcirculatory abnormalities is associated with poor outcome [3, 4].

In recent years, methods such as sidestream dark field imaging (SDF) [6] or incident dark field illumination imaging (IDF) [7] were developed to allow a direct observation of the microcirculation at the bedside. Directly monitoring the microcirculation is a powerful diagnostic tool and may help to better understand the individual problems of patients and the effects of haemodynamic therapy in the critical care setting [8]. Based on the availability of microcirculatory monitoring at the bedside and accumulating evidence, treatment decisions may be made on the basis of microvascular parameters in the near future [9, 10].

A major shortcoming constraining the widespread evaluation of the microcirculation at the bedside is the prolonged time necessary for the quantitative analysis. The current gold standard and method of choice is a time-consuming manual analysis that includes plotting of each vessel and quantification of flow in every individual vessel in a video performed by an experienced user [11]. A software conducting this analysis automatically could help to establish the microcirculation as a tool for “point-of-care” diagnosis and decision-making.

The aim of this study was to compare a software for an automatic analysis of key microcirculatory variables, such as total vessel density (TVD), perfused vessel density (PVD) and proportion of perfused vessels (PPV), with a manual analysis, which is the current gold standard [11]. An additional intention was to correlate the results of these two methods.

## Methods

### Study design

Ten sheep were anaesthetized and instrumented as described below. After reaching shock time point and the respective measurements, data acquisition for the present study ends, and the sheep were randomised for an interventional study of haemorrhagic shock, whose data is not part of the present analysis. At the end of that study, the sheep were killed with an intravenous injection of 4 mg/kg propofol and of 200 mL potassium chloride solution (7.45%).

### Anaesthesia and instrumentation

General anaesthesia was induced in ten female sheep (species *Ovis orientalis aries*) by intramuscular bolus injection of 0.3 mg·kg^−1^ midazolame and 10 mg·kg^−1^ S-ketamine. Following endotracheal intubation, balanced anaesthesia was maintained by inhalational isoflurane with an expiratory fraction of 1.2 vol% as well as a continuous infusion of 0.3 mg·kg^−1^·h^−1^ midazolame and 1 mg·kg^−1^·h^−1^ S-ketamine. The sheep were ventilated targeting an end-tidal carbon dioxide partial pressure of 35 ± 5 mmHg.

A pulse contour cardiac output catheter (5-Fr PiCCO™ catheter, Pulsion Medical Systems, Munich, Germany) was placed in the left femoral artery to obtain haemodynamic variables. A central venous line was inserted into the right jugular vein to administer drugs and to conduct thermodilution as well as a 7.5-Fr catheter in the left jugular vein for blood withdrawal. A Foley catheter was placed in the urinary bladder.

Afterwards, the sheep were turned into prone position and were allowed to recover for a period of 30 min before the experimental protocol was started.

### Experimental protocol

Following baseline measurements (“baseline” time point), 3 × 10 mL blood per kilogram body weight was withdrawn stepwise over a period of 5 min each. After each of the three blood withdrawals, there was a recovery period of 30 min. The resulting 30 mL per kg body weight of withdrawn blood equals approximately 50–60% of the total blood volume in the sheep [12]. If mean arterial pressure (MAP) decreased below 30 mmHg during blood withdrawal, the current step of withdrawal was stopped immediately resulting in less than 10 mL per kg body weight for the individual withdrawal.

After 3 cycles of blood withdrawal, each followed by 30 min of recovery time, shock time was defined after the last recovery time. At shock time point, haemodynamic and microcirculatory measurements were performed.

### Measurements

At both time points (baseline and shock), systemic haemodynamic variables and conjunctival microcirculation were measured and arterial blood samples were obtained for the blood gas analysis.

Haemodynamic measurements comprised MAP, stroke volume index (SVI) and cardiac index (CI). SVI and CI were obtained by threefold bolus thermodilution using the PiCCO™ system. The mean of the three measurements was documented. Also, the heart rate was read from the haemodynamic monitoring tool.

Conjunctival microcirculation was measured in five different conjunctival positions at both measurement time points. Measurements were conducted using an IDF camera (CytoCam™, Braedius Medical BV, Huizen, The Netherlands). The obtained videos were reviewed for quality according to recommendations by Massey et al. [13] and discarded if necessary.

The remaining high-quality videos were analysed (1) by an experienced user blinded for the study protocol using a manual analysis software (AVA software version 3.2, Microvision Medical, Amsterdam, The Netherlands) and (2) a software including an automatic video analysis of microcirculatory videos (AVA software version 4.2, Microvision Medical, Amsterdam, The Netherlands).

With both programs, the TVD, PVD and the PPV were determined in each video according to an independent expert consensus conference to characterise microcirculatory perfusion [11].

Manual analysis with AVA software version 3.2 comprised the manual drawing of each vessel and assigning a diameter and flow score to each vessel and has been described thoroughly before [14]. After the manual processing, the AVA 3.2 software calculated the above-mentioned parameters. In the AVA software version 4.2, the software conducts the process of detecting the vessels and flow automatically. As the consensus conference recommended including only vessels with a diameter of less than 20 μm in the microcirculatory analysis, this threshold was used in both analysis methods.

### Statistical analysis

Statistical analysis was performed with IBM SPSS statistics software version 22 (IBM, Armonk, New York, USA). All data are presented as mean with standard deviation, unless otherwise stated.

Variables were tested to confirm the equality of variances by Levene’s test, and Kolmogorov-Smirnov test was used to confirm normal distribution. For comparisons between time points, paired sample *t* test was used. Asymptotic two-sided *p* values smaller than 0.05 were assumed as statistically significant.

For the comparison of manual and automatic analyses of sheep, conjunctival microcirculation intraclass correlation coefficient (ICC) was determined [15]. The ICC for normally distributed, continuous values is presented with 95% confidence intervals as a measure of dispersion. Values below 0.40 are considered as “poor” agreement, between 0.40 and 0.59 as “fair” agreement, between 0.60 and 0.74 as “good” agreement and for greater than 0.74, the level of agreement is “excellent” as suggested by Cicchetti [16].

In addition, the method suggested by Bland and Altman [17] was used to assess the agreement between the two analysis methods. According to Bland and Altman, the mean difference of the two values (manual and automatic) for each video was plotted against the average of those two values. The mean bias (95% confidence interval) was calculated as well as the limits of agreement (LOA) as 1.96-fold of the standard deviation of the mean bias.

## Results

### Haemodynamic variables and arterial blood gas analysis

24.3 ± 4.5 mL/kg of blood was withdrawn. In seven sheep, blood withdrawal was stopped due to predefined safety measures. Withdrawal of blood resulted in a decrease in MAP, CI and SVI and an increase in HR from baseline to shock time point. In addition, an increase in arterial lactate concentration as well as a reduction of arterial haemoglobin concentration was found (Table 1).

**Table 1:** Haemodynamic variables and arterial blood gas analysis

Variable	Baseline (*n* = 10)	Shock (*n* = 10)	*p* value
MAP [mmHg]	87 ± 11	40 ± 7	<0.001^§^
HR [bpm]	72 ± 9	87 ± 11	0.003^§^
CI [L min^−1^·m^−2^]	2.9 ± 0.9	1.8 ± 0.5	<0.001^§^
SVI [mL·m^−2^]	38 ± 14	20 ± 5	0.001^§^
Hb [g·dL^−1^]	8.6 ± 0.7	8.0 ± 0.5	0.003^§^
Lactate [mmol·L^−1^]	0.9 ± 0.3	2.0 ± 0.6	0.001^§^

All values are presented as mean ± standard deviation. Lactate = arterial lactate concentration

*MAP* mean arterial pressure, *HR* heart rate, *CI* cardiac index, *SVI* stroke volume index, *Hb* arterial haemoglobin concentration

^§^Significant difference between baseline and shock

### Microcirculatory variables at baseline and in haemorrhagic shock

Of the 100 videos taken of sheep conjunctival microcirculation, 85 met the predefined quality criteria [13]. Microcirculatory variables were examined regarding differences between baseline and shock for each of the two analysis methods (Table 2). Manual analysis showed a significant decrease in PVD and a significant reduction of PPV in shock compared to baseline, while TVD remained constant. Contrarily, no statistically significant differences could be demonstrated between baseline and shock for any of the variables with the automatic analysis (Additional files 1 and 2).

**Table 2:** Microcirculatory variables

Analysis method	Variable	Baseline (*n* = 10)	Shock (*n* = 10)	*p* value
Manual analysis	TVD [mm*mm^−2^]	17.8 ± 4.2	17.8 ± 3.8	0.993
	PVD [mm*mm^−2^]	15.6 ± 4.6	11.5 ± 6.5	0.041^§^
	PPV [%]	85.9 ± 11.8	62.7 ± 29.6	0.017^§^
Automatic analysis	TVD [mm*mm^−2^]	10.6 ± 1.4	11.0 ± 1.9	0.460
	PVD [mm*mm^−2^]	10.2 ± 1.4	10.8 ± 1.9	0.330
	PPV [%]	96.1 ± 6.	97.9 ± 3.7	0.372

All values are presented as mean ± standard deviation

*TVD* total vessel density, *PVD* perfused vessel density, *PPV* proportion of perfused vessels

^§^Significant difference between baseline and shock

### Results of intraclass correlation coefficient and Bland-Altman analysis

The ICC, calculated for the complete data set (*n* = 85) and for the subgroups baseline (*n* = 41) and shock (*n* = 44), showed a poor agreement [16] for all examined variables (Table 3).

**Table 3:** Intraclass correlation coefficient between manual and automatic analyses

Variable	Data set	Number	ICC [95% CI]	Agreement
TVD [mm*mm^−2^]	All videos	85	−0.267 [−0.949–0.176]	Poor
	Baseline	41	−0.069 [−1.01–0.430]	Poor
	Shock	44	−0.568 [−1.874–0.144]	Poor
PVD [mm*mm^−2^]	All videos	85	−0.219 [−0.875–0.208]	Poor
	Baseline	41	−0.013 [−0.899–0.460]	Poor
	Shock	44	−0.379 [−1.538–0.247]	Poor
PPV [%]	All videos	85	−0.074 [− 0.651–0.302]	Poor
	Baseline	41	0.274 [− 0.361–0.613]	Poor
	Shock	44	−0.193 [−1.187–0.349]	Poor

Agreement as suggested by Cicchetti [15] (values below 0.40 are considered as “poor” agreement, between 0.40 and 0.59 as “fair” agreement, between 0.60 and 0.74 as “good” agreement and for greater 0.74, the level of agreement is “excellent”)

*ICC* intraclass correlation coefficient, *CI* confidence interval, *TVD* total vessel density, *PVD* perfused vessel density, *PPV* proportion of perfused vessels

**Fig. 1:**
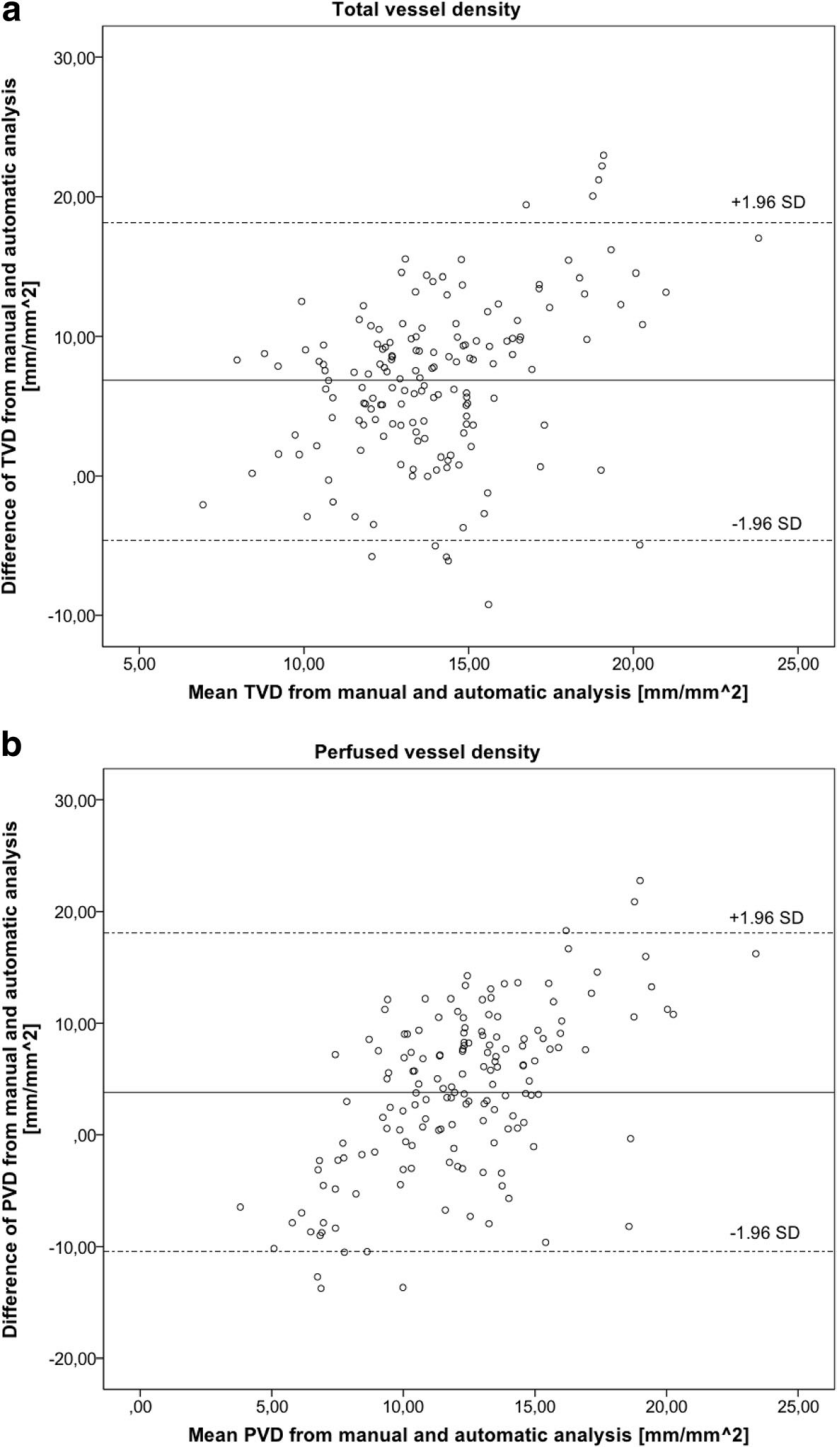
Bland-Altman plots for **a** total vessel density and **b** perfused vessel density (each *n* = 85). Bland and Altman recommended plotting the mean of two measurement methods against the difference of both [17]. *Continuous line* represents the mean difference whereas *upper and lower dashed lines* represent the limits of agreement (LOA) (equivalent to ±1.96 SD of mean difference). Bland-Altman plots have to be inspected visually. Mean difference represents the mean bias between measurements. LOA are a measure of dispersion between both methods. The evaluator has to decide whether these values are acceptable in the context of the measured variable. *TVD* total vessel density, *PVD* perfused vessel density, *SD* standard deviation, *LOA* limits of agreement

In addition, the Bland-Altman analysis revealed a bias between manual and automatic analyses for all tested variables with wide LOA (Table 4). Figure 1 demonstrates the respective Bland-Altman plots for TVD and PVD (*n* = 85).

**Table 4:** Bland-Altman analysis between manual and automatic analyses

Variable	Data set	Number	Mean bias [95% CI]	LOA
TVD [mm*mm^−2^]	All videos	85	7.44 [6.12–8.77]	−4.57–19.45
	Baseline	41	7.66 [5.66–9.67]	−4.79–20.11
	Shock	44	7.24 [5.42–9.06]	−4.50–18.98
PVD [mm*mm^−2^]	All videos	85	3.37 [1.61–5.12]	−12.58–19.32
	Baseline	41	5.61 [3.45–7.78]	−7.84–19.06
	Shock	44	1.27 [−1.38–3.93]	−15.84–18.38
PPV [%]	All videos	85	−23.09 [−29.66–(−16.51)]	−82.83–36.65
	Baseline	41	−11.62 [−17.34–(−5.91)]	−47.12–23.88
	Shock	44	−33.77 [−44.60–(−22.94)]	−130.57–36.03

*LOA* limits of agreement, *TVD* total vessel density, *PVD* perfused vessel density, *PPV* proportion of perfused vessels

## Discussion

The main results of the present study are that ovine haemorrhagic shock induced by blood withdrawal caused a reduction in PVD and PPV if analysis of microcirculation was conducted manually (with AVA software version 3.2). Automatic analysis (by AVA software version 4.2) was not able to reproduce the findings of the gold standard method. Accordingly, there was a wide variation of values, and Bland-Altman analysis as well as ICC revealed a poor correlation between manual and automatic analyses.

A reliable automatic analysis of microcirculation would be associated with multiple advantages. First, this method would be markedly faster than manual analysis thus allowing bedside application. Based upon our own experiences, automatic analysis by AVA software version 4.2 takes approximately 3 to 4 min while manual analysis by an experienced user takes about 20 min per video. Second, based on the faster analysis at the bedside, monitoring of the microcirculation would become applicable for immediate therapeutic decisions. Third, the analysis would be independent of the user’s experience concerning the technical analysis. For these reasons, a reliable automatic analysis of the microcirculation would represent a major advantage for its clinical impact.

In the present study, macrohaemodynamic measurements after blood withdrawal revealed the typical signs of hypovolaemic shock, namely decreases in MAP, CI and SVI accompanied with an increase in heart rate. Simultaneously, lactate levels increased which might have been caused by a deranged microcirculation due to the induction of haemorrhagic shock on the one hand, but also by catecholamine release, mitochondrial and cellular dysfunction on the other hand. The decrease in haemoglobin concentration may best be explained by haemodilution due to endogenous recruitment of interstitial fluid into the intravascular compartment.

Manual analysis of the microcirculation showed results concordant with macrohaemodynamic features of hypovolaemic shock. TVD remained constant during the induction of haemorrhagic shock, and PVD and PPV were reduced. These observations support those of other investigators in clinical and preclinical studies of microcirculatory changes in haemorrhagic shock [18, 19]. The automatic analysis, however, was not able to demonstrate these characteristic microcirculatory changes of haemorrhagic shock.

Examination of the Bland-Altman plots revealed a bias in the same variables whose dimension was high. Furthermore, LOA were too wide for all variables. According to Bland and Altman, these signs not only indicate a constant bias between the two methods but also a high variation making a correlation between them unlikely [17]. Accordingly, the comparison of the two methods evaluated by ICC revealed a poor agreement in all TVD, PVD and PPV.

Microcirculatory changes in haemorrhagic shock potentially could have influenced the correlation analysis. There is a risk that vessels, which were intermittently (e.g. large plasma gaps) or very slowly perfused by red blood cells, are better recognised by an experienced operator than by an automatic analysis software. Therefore, the correlation at baseline (when perfusion is supposed to be physiological) might be better than in haemorrhagic shock, where low flow vessels are more likely to be found. To exclude this suspicion, the agreement between manual and automatic analyses was investigated separately for each time point (baseline and shock). Notably, no correlation or agreement between the two analysis methods was found when separating the values for baseline and shock. As a consequence, an influence of the pathophysiological changes during haemorrhagic shock on the correlation seems to be unlikely.

**Fig. 2:**
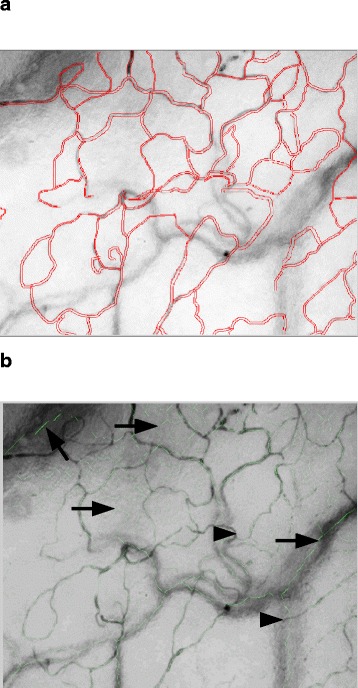
Screenshot of capillary networks analysed **a** manually and **b** automatically. Screenshot of the same video: **a** analysed manually by an experienced user with the AVA software version 3.2 and **b** analysed automatically by the AVA software version 4.2. Borders of vessels in panel **a** are coloured *red*, and centre lines of vessels in panel **b** are coloured *lime green. Marks* in panel **b** show examples of differences from automatic to manual analysis: *Arrowheads* = no vessel detected compared to manual analysis; *arrows* = more vessels detected as by manual analysis

The poor correlation between both analyses could reasonably be attributed to shortcomings of the analysis algorithm for vessel detection in the automatic analysis as illustrated in Fig. 2. Figure 2 shows screenshots of the capillary networks drawn by automatic analysis and manually by an experienced user in a corresponding video of ovine conjunctival microcirculation. The automatic analysis algorithm was not able to differentiate between different focus depths meaning that the algorithm detects vessels, which were located outside the focal plane, and, in contrast, would not be “drawn in” by an experienced investigator using manual analysis software. This misinterpretation by automatic analysis leads to different values for vessel-density-dependent variables compared to manual analysis. Further improvement of automatic analysis software will primarily have to challenge the shortcomings of vessel detection to improve overall quality of analysis results.

Parameters that describe flow conditions of the microcirculation are important as they provide information about diffusion and convection [9]. Following the consensus recommendations, evaluating the microcirculation should include a flow index [11]. An overview of flow conditions in the microcirculation is, for example, provided by the semi-quantitative microvascular flow index (MFI) by classifying microvascular flow into different categories (no, intermittent, sluggish or continuous flow) [20]. The automatic analysis provides no flow-related results except PPV and PVD, which are estimates of the flow-dependent functional capillary density, but provide no information about the flow itself. Thus, it was not possible to carry out a comparison of variables describing flow between manual analysis and automatic analysis. The missing detection of different flow states by automatic analysis would also be a likely explanation for the lack of changes in PVD and PPV following haemorrhage as detailed above. In the flow-independent parameter TVD (which simply detects all visible vessels), both analysis methods revealed no changes between baseline and shock. Manual analysis was able to detect changes in the flow-dependent variables PVD and PPV whereas automatic analysis failed to detect these.

The present study has some limitations that need to be mentioned. As videos were obtained in an animal model of haemorrhagic shock, results may not apply to human microcirculatory analysis. In addition, videos of ovine conjunctival microcirculation were investigated, whereas the sublingual region is usually the area of choice for the clinical examination of human microcirculation (especially for studying abnormalities in sepsis or haemorrhagic shock) [2, 11, 19]. The reason for choosing the sublingual region is, on the one hand, its easy accessibility and, on the other hand, its blood perfusion is based on proximity to the central arteries. As the same arguments regarding vascular anatomy and accessibility apply to the conjunctival microcirculation, it may also be a valuable target. The good correlation between sublingual and conjunctival microcirculation has recently been demonstrated in an experimental study by Yin et al. [21]. Based on our own experience, video quality of ovine conjunctival microcirculation videos is superior to the quality of videos derived from ovine sublingual microcirculation due to anatomical conditions such as a long floppy tongue vs. large eyeballs with easy access. Thus, we decided to perform a comparison between manual and automatic analyses using high-quality conjunctival videos. It may be possible that automatic analysis works better with sublingual videos of microcirculation. Also, the AVA software version 4.2 was developed to automatically analyse videos obtained by SDF imaging technique. However, as microcirculatory videos obtained by IDF technique provide better image quality [22, 23] and can be converted to and opened in AVA software version 4.2, analysis results should be at least as good as with videos from SDF cameras. At last, it is unclear if the results of the present study can be transferred to pathophysiological conditions other than haemorrhagic shock as we only examined microcirculation during the induction of haemorrhage. Further investigations are needed to address these limitations.

## Conclusions

Characteristic changes in microcirculation during ovine haemorrhagic shock could not be reproduced by automatic analysis with the AVA 4.2 software. No flow-quantifying variable was given by automatic analysis, and there was only a poor correlation between automatic analysis and the current gold standard (manual analysis). Therefore, the automatic analysis with the AVA 4.2 software cannot be recommended at present. Further improvements in the detection algorithm are necessary, before an automatic analysis could be re-evaluated for the evaluation of microcirculation in research or clinical routine.

## Additional files

Additional file 1: Raw data of blood withdrawal, haemodynamics, blood gas analysis and microcirculatory analysis. (XLSX 10 kb)
Additional file 2: Raw data of microcirculatory analysis of individual videos. (XLSX 14 kb)

## Abbreviations

CI: cardiac index
HR: heart rate
ICC: intraclass correlation coefficient
IDF: incident dark field
LOA: limits of agreement
MAP: mean arterial pressure
PPV: proportion of perfused vessels
PVD: perfused vessel density
SDF: sidestream dark field
SVI: stroke volume index
TVD: total vessel density
